# Supplementary material for: Kinetic characteristics of propofol-induced inhibition of electron-transfer chain and fatty acid oxidation in human and rodent skeletal and cardiac muscles
Source: PLoS One. 2019 Oct 4;14(10):e0217254. doi: 10.1371/journal.pone.0217254 (PMC6777831; doi:10.1371/journal.pone.0217254)
Supplement: S1 File — (DOCX) [file pone.0217254.s001.docx]

**Supplementary Appendix**

To: Urban T et al.: *Kinetic characteristics of propofol-induced inhibition of electron-transfer chain and fatty acid oxidation in human and rodent skeletal and cardiac muscles*

S1.0 Supplemetary methods

S1.1 Study subject characteristics

Table A. Human tissue donor characteristics

| Brain-dead organ donor | Age | Sex | Significant co-morbidities and medication |
| --- | --- | --- | --- |
| #1 | 71 | Female | Haemorhagic stroke; Hypertension, Asthma; salbutamol, urapidil |
| #2 | 63 | Female | TBI Hypertension, hyperlipidaemia, stable coronary heart disease |
| #3 | 50 | Male | TBI; Noradrenaline |
| #4 | 40 | Female | TBI; Stable coronary heart disease, COPD, steroids, noradrenaline |
| #5 | 26 | Male | SAH; Fatty liver, upper respiratory infection, pancreatic cyst |
| #6 | 64 | Female | Hypoxic brain injury; Pulmonary embolism, noradrenaline |
| #7 | 48 | Female | TBI; Hypertension, diabetes mellitus; noradrenaline, vasopressin, dobutamine, furosemide |
| #8 | 67 | Female | TBI Hypertension, chronic kidney disease, craniotomy, noradrenaline |
| #9 | 76 | Male | Stroke; Hypertension, aortic stentgraft; ramiprile tiapridal, noradrenaline |
| #10 | 60 | Male | TBI; Noradrenaline |

S1.2 Step-by-step protocol for high resolution respirometry for human heart homogenates

(adopted from: Krajcova et al. *High resolution respirometry to assess function of mitochondria in native homogenates of human heart muscle [2019 under review]*)

1. Firstly, wash properly all tubes, scissors, forceps, teflon pestles and glassware (including Dounce Tissue homogenizer and glass pestles) with 70% ethanol and tap water and keep them on ice to cool to 0°C. Immediatelly, before use, rinse the instruments once again using distilled water and thoroughly dry them with clean gauze. Glass and teflon grinders were used from Micro Tissue Grinder Kit (Wheaton^TM^, Millville, USA)
2. Prepare hand-stitched cornet from polyamide mesh (parameters: loop size 335 µm, fibre diameter 120 µm, material: 100% polyamide; SILK & PROGRESS s.r.o., Czech Republic)


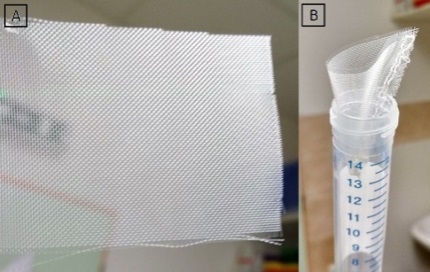


Figure A. Hand-sawn cornet.

1. Immediately after dissection, insert muscle biopsy sample from heart appendage/ventricle into plastic 50 ml Falcon tube with 10 ml of cold BIOPS and transfer it to the lab on ice in the closed plastic storage box.
2. Firstly, place biopsy sample on piece of parafilm on ice.
3. Remove fat, connective tissue and blood vessels from heart muscle tissue using clean pre-cold scissors and forceps
4. Check the sample under the microscope. Place the sample on the piece of parafilm/or lid of Petri dish on the bottom plate of Petri dish (60 mm; polystyrene) filled with ice and put it under the microscope. Check the sample and remove remaining fat and connective tissue if necessary.
5. Dry the sample gently by piece of gauze to remove residual BIOPS and blood (  ̴̴ 5 seconds)
6. Weight the sample on the analytical scale placed on parafilm
7. Dissect the sample with scissors into fine fragments. During this step, the muscle has to be placed on the parafilm on ice.
8. Transfer carefully the pieces of muscle into the pre-cold Wheaton^TM^ 1 mL Dounce Tissue Grinder and dilute in ratio: 100 mg of the muscle per 1 mL of MiR05 medium to obtain 10% muscle homogenate
9. Homogenize gently the muscle pieces in 1 mL Dounce Tissue Grinder by moving the pre-chilled glass pestle (Loose) up and down (10-12 strokes). During the procedure, Grinder has to be placed on ice.


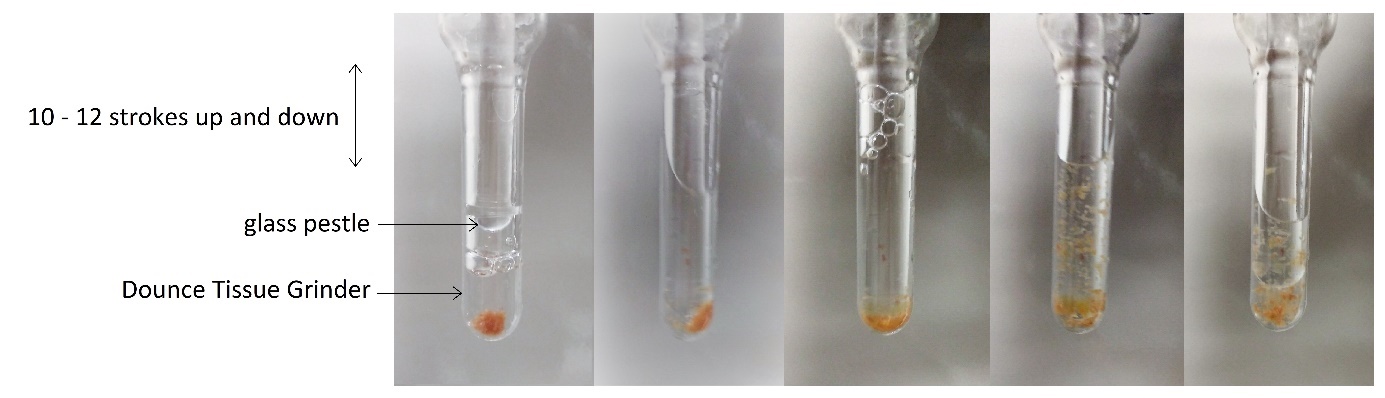


Figure B. Manual homogenization with 10 – 12 up and down. The process must be done **on ice**.

1. Wash the pre-cold shaft with PTFE pestle from Wheaton^TM^ 2mL Potter-Elvehjem Tissue Grinder by distilled water and dry the pestle by sterile gauze. Fix the shaft into motor-driven homogenizer (HEi-Torque Value 100, Heidolph, Germany). Set the speed to 750 rpmi.
2. **Critical step.** Insert the glass tube of 1 mL Dounce Tissue Grinder (with muscle pieces floating in MiR05 inside) into plastic 50 mL Falcon tube with crushed ice (diluted with small amount of tap water). Be careful to leave the upper part of glass tube protruding out of the crushed ice. Immerse the PTFE pestle into the glass tube of 1 mL Dounce Tissue Grinder and turn on the motor-driven homogenizer. Whilst speed is getting from 0 to 750 rpmi, slowly move the pestle down to touch the bottom of glass tube. Gently move up and down 5-6 times whilst the pestle is still inserted in the homogenate (this helps to avoid creating bubbles inside under pressure)


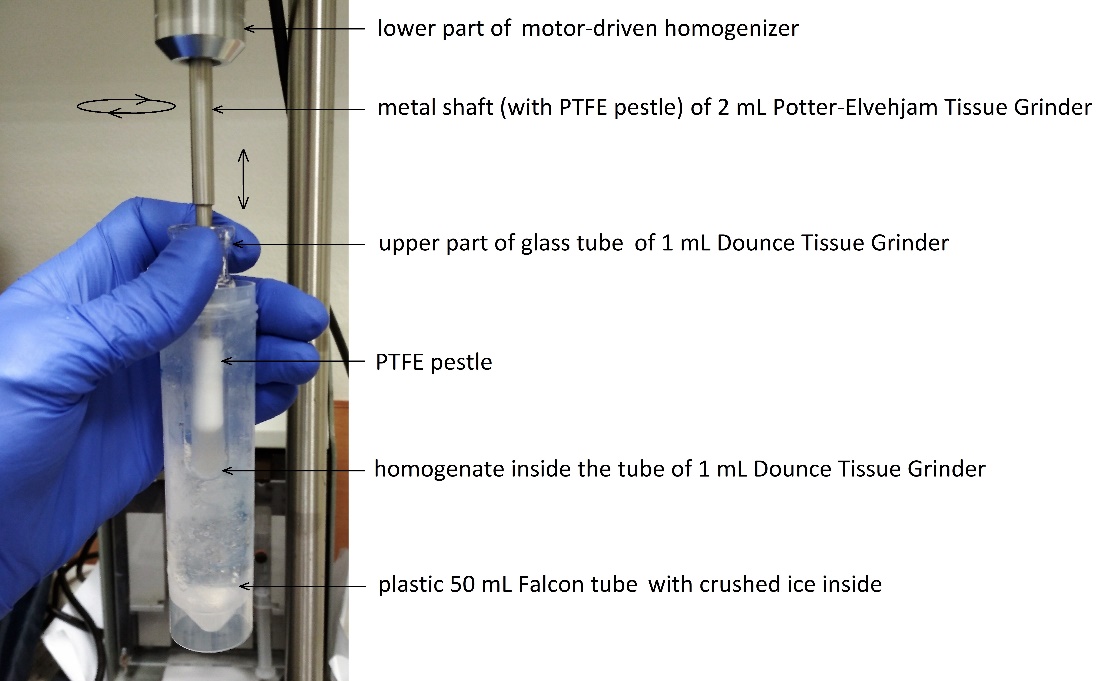
Figure C. Motor-driven homogenization (750 rpmi) simultaneously with manual 5-6 strokes up and down

1. In the end of homogenization process, slowly pull down the glass tube (of 1 mL Dounce Tissue Grinder) in tube with crushed ice from the pestle and simultaneously gradually slow down the speed of motor-driven homogenizer by turning the wheel (back to 0 rpmi).
2. Dilute 10 % homogenate with MiR05 3 times to get the final concentration of 2.5% solution.
3. Repeat step m: motor-driven homogenization (750 rpmi) with simultaneous manual 3-4 (only!) strokes up and down. You will get crude 2.5% homogenate.
4. Filter crude homogenate through polyamide technical screen into pre-cold plastic 12 mL Falcon tube or 2 mL Eppendorf tube.
5. Store the Falcon tube or Eppendorf tube with filtered homogenate on ice until the polarographic experiment will be performed (**do not leave the homogenate on ice more than 1 hour**; in case that measurement is not performed until 1 hour after homogenization **it is necessary** to prepare a new fresh homogenate).
6. After calibration of Oxygraph-2k, pipette 200 µL of filtered homogenate inside the opened chamber (use pipette with tips of 1 mL volume).
7. Carefully close the chamber without creating bubbles inside and perform experiment using high-resolution respirometry
8. Inject the agents (substrates, uncouplers or inhibitors) according to SUIT protocol into the chamber with Hamilton syringe.

S2.0 Supplementary results.

S2.1 Spectrophotometry analysis of isolated complexes


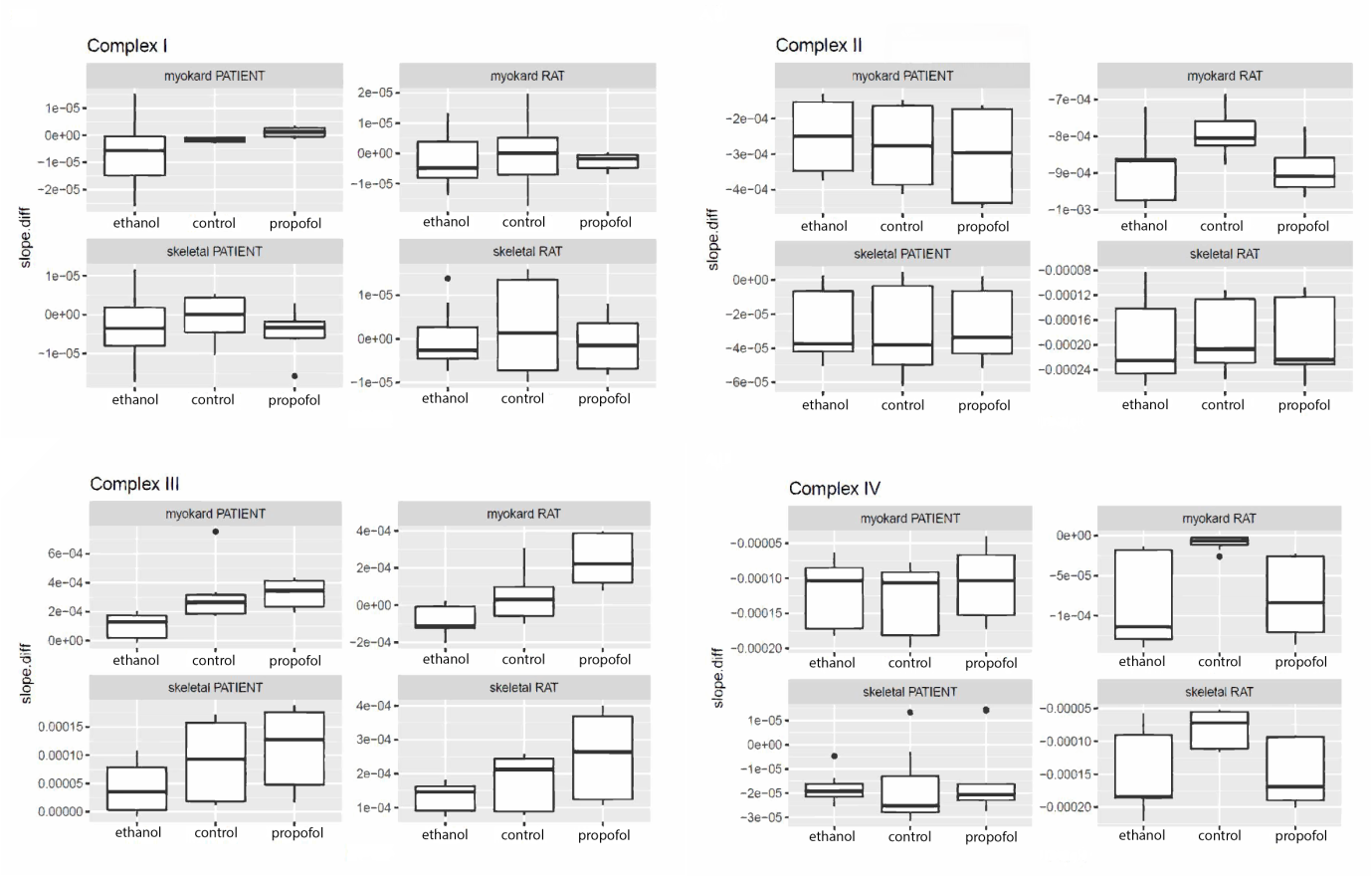


Figure D. Results of spectrophotometry analysis of respiratory complexes in the presence of propofol (right), vehicle (ethanol, left) or fresh media (middle column).
